# Gold-Nanorod-Assisted Live Cell Nuclear Imaging Based on Near-Infrared II Dark-Field Microscopy

**DOI:** 10.3390/biology12111391

**Published:** 2023-10-31

**Authors:** Yifeng Shi, Shiyi Peng, Zhongyu Huang, Zhe Feng, Wen Liu, Jun Qian, Weidong Zhou

**Affiliations:** 1 Key Laboratory of Optical Information Detection and Display Technology of Zhejiang, Zhejiang Normal University, Jinhua 321004, China; shiyifeng@zjnu.edu.cn (Y.S.); hzhongyu@zjnu.edu.cn (Z.H.); wdzhou@zjnu.cn (W.Z.); 2 State Key Laboratory of Modern Optical Instrumentations, Centre for Optical and Electromagnetic Research, College of Optical Science and Engineering, International Research Center for Advanced Photonics, Zhejiang University, Hangzhou 310058, China; 12330003@zju.edu.cn (S.P.); zhefeng@zju.edu.cn (Z.F.)

**Keywords:** dark-field microscopy, gold nanorods, near-infrared second window, live cell nuclear imaging

## Abstract

**Simple Summary:**

Colorectal cancer cells exhibited superior dark-field imaging results in the near-infrared II (NIR-II) wavelength range (900–1880 nm) compared to the visible light region. Subsequently, we synthesized gold nanorods (GNRs) for dark-field scattering imaging of colorectal cancer cells in the NIR-II spectrum. The results demonstrated that imaging with GNRs significantly improved the signal-to-background ratio (SBR) and showed enhanced performance, particularly in the 1425–1475 nm wavelength range. Finally, we conducted dark-field imaging of cell nuclei in the NIR-II range utilizing GNRs for specific labeling of colorectal cancer cell nuclei. The resulting nuclear images were highly accurate in localization and exhibited higher SBR compared to non-specifically-labeled cell imaging.

**Abstract:**

Dark-field microscopy offers several advantages, including high image contrast, minimal cell damage, and the absence of photobleaching of nanoprobes, which make it highly advantageous for cell imaging. The NIR-II window has emerged as a prominent research focus in optical imaging in recent years, with its low autofluorescence background in biological samples and high imaging SBR. In this study, we initially compared dark-field imaging results of colorectal cancer cells in both visible and NIR-II wavelengths, confirming the superior performance of NIR-II imaging. Subsequently, we synthesized gold nanorods with localized surface plasmon resonance (LSPR) absorption peaks in the NIR-II window. After bio-compatible modification, we non-specifically labeled colorectal cancer cells for NIR-II dark-field scattering imaging. The imaging results revealed a sixfold increase in SBR, especially in the 1425–1475 nm wavelength range. Finally, we applied this imaging system to perform dark-field imaging of cell nuclei in the NIR-II region and used GNRs for specific nuclear labeling in colorectal cancer cells. The resulting images exhibited higher SBR than non-specifically-labeled cell imaging, and the probe’s labeling was precise, confirming the potential application of this system in photothermal therapy and drug delivery for cancer cells.

## 1. Introduction

Biological imaging is a crucial component of modern medicine. Optical imaging techniques offer advantages, such as radiation-free imaging, high spatial and temporal resolution, low photon damage, and real-time imaging capabilities, when compared to traditional imaging methods. Therefore, optical biological imaging techniques have unique advantages in many applications. The structure and function of biological organisms are fundamentally based on cells, particularly the cell nucleus. Various optical microscopy techniques are commonly used for cellular imaging, including bright-field imaging, dark-field imaging, and fluorescence imaging [1,2]. Among these, bright-field imaging is the most basic microscopy technique, but it often exhibits low image contrast, making it more suitable for samples with strong absorption. As research has advanced in imaging systems, more sophisticated microscopy techniques, such as fluorescence microscopy, have emerged as essential tools in modern biological research. Fluorescence microscopy [3,4] is highly sensitive and non-invasive, offering high spatial and temporal resolution for imaging live cells and monitoring dynamic cellular processes [5,6]. However, when using fluorescence microscopy, higher laser excitation power is often required, and common dyes used during imaging are susceptible to photobleaching; additionally, the resolution can be limited by the autofluorescence of biological samples. Therefore, for cellular imaging, bright-field and dark-field microscopy are commonly employed, with dark-field microscopy being particularly advantageous. Dark-field microscopy (DFM) [7] is a specialized optical microscopy technique that can replace more complex or expensive microscopy methods in certain applications. DFM allows rapid identification of nanoscale particles, microscale cells, and tissues [8,9,10] in biological samples and environmental samples, such as wastewater, soil, sediments, etc. [11,12] and provides an effective means for rapid, efficient, cost-effective, and non-destructive imaging of nanoscale particles. DFM illuminates the sample with oblique light, typically using an illumination aperture outside the NA of the objective lens. A central stop or mask is added to the system to block the central light beam from the light source, causing the incident light to be angled onto the sample. Consequently, DFM imaging yields a dark field of view with higher image contrast. It is important to note that DFM does not inherently enhance image resolution but instead improves the contrast of image edges that are obscured by bright-field illumination.

Typically, light in the range of 700–2500 nm is referred to as near-infrared (NIR) light. NIR light is characterized by low scattering in biological tissues and low autofluorescence, making it suitable for biological imaging and yielding images with a higher signal-to-background ratio (SBR) [13,14]. NIR light can be further divided into two windows: the first near-infrared window (NIR-I, 760–900 nm) and the second near-infrared window (NIR-II, 900–1880 nm). Among these, the NIR-II window has two notable characteristics: first, it exhibits significant water absorption, which reduces background noise from scattered signal photons in wide-field imaging techniques; second, it offers low scattering, enabling deeper imaging within living organisms [15]. As such, the NIR-II window provides advantages in terms of imaging depth and image SBR. Hong et al. [16] investigated the absorption spectrum of water at various wavelengths and found that water exhibited absorption peaks at 970 nm, 1200 nm, 1450 nm, and beyond 1800 nm. Among these peaks, the absorption at 1450 nm was particularly strong, significantly affecting imaging quality. However, recent research has shown that the absorption characteristics of this wavelength range can be utilized to improve imaging quality. Feng et al. [17] simulate the photon propagation in the NIR region, confirming the positive contribution of moderate light absorption by water in intravital imaging.

Nanomaterials, owing to their diverse types, wide spectral coverage, chemical stability, and resistance to photobleaching, are frequently employed in biological imaging. Researchers are actively engaged in the development and preparation of NIR-II nanoprobes to further enhance imaging quality. The nanomaterials commonly used can be divided into inorganic materials, such as gold nanorods, carbon nanotube [18], quantum dot [19], rare-earth-doped nanoparticles [20], etc., and organic materials, mainly conjugated polymer [21]. Among these, gold nanorods [22] are rod-shaped nanostructures, with dimensions typically below a few hundred nanometers, composed primarily of gold. GNRs offer stability in chemical properties, tunable scattering peaks [23], diverse synthesis and modification methods [24], flexible size control, and easy conjugation with other molecules to alter their physical and chemical properties, making them extensively researched and applied. In particular, GNRs exhibit unique LSPR properties, resulting from the interaction between electrons and electromagnetic waves at the nanoscale. When the wavelength of incident light is approximately ten times the size of the nanorods, the surface electrons undergo collective oscillations, causing most of the incident photons with frequencies matching the collective oscillation frequency of valence electrons to be absorbed and scattered. The presence of LSPR peaks in the extinction spectrum is a prominent feature at the macroscopic level. These LSPR peaks indicate enhanced absorption and scattering properties of GNRs, making them advantageous for various applications, particularly in the field of biological imaging [25]. GNRs possess a substantial scattering cross-section, and variations in their aspect ratios result in different longitudinal LSPR absorption peaks, enabling them to scatter light at different wavelengths [26]. When GNRs scatter light in the near-infrared range, they offer significant advantages for biological imaging. The enhanced scattering capability of GNRs can be harnessed for dark-field microscopy of cell nuclei.

In this study, taking advantage of the NIR-II window, we optimized a dark-field imaging system and employed GNRs as probes for dark-field imaging of cell nuclei. Initially, we observed that compared to the visible light spectrum, NIR-II imaging offered reduced background noise, clearer cell contours, and increased contrast, particularly with increasing wavelength. However, cell scattering in the NIR-II range was relatively low, resulting in lower light signal intensity. To further enhance imaging quality, we introduced gold nanoprobes to enhance cell scattering and, consequently, signal intensity. We synthesized GNRs using a one-pot method, applied biocompatible polymer modification, and non-specifically labeled live cells for use in NIR-II dark-field scattering imaging. However, polymer-modified GNRs could only enter the cell cytoplasm and could not access the cell nucleus. To image cell nuclei, we modified GNRs with membrane-penetrating proteins and nuclear localization proteins for specific nuclear labeling, ultimately obtaining well-defined nuclear boundary images. The specificity of the probe’s labeling closely matched that of nuclear-specific dyes. This demonstrates that our research holds practical significance and offers potential applications in the treatment of cancer cells and drug delivery within the realm of biomedicine.

## 2. Materials and Methods

### 2.1. Experimental Reagents

The reagents for GNR preparation were chloroauric acid (HAuCl_4_), silver nitrate (AgNO_3_), cetyltrimethylammonium bromide (CTAB), Hydroquinone (HQ), and sodiumborohydride (NaBH_4_), which were all purchased from Sigma-Aldrich (St. Louis, MO, USA).

The reagents for polymer electrolyte multilayer coating of GNRs were Poly(3,4-ethylenedioxythiophene)-polystyrene sulfonate (PSS, MW = 240,000) purchased from Polysciences, Inc. (Warrington, PA, USA); Poly (allylamine hydrochloride) (PAH) purchased from Beijing Huaweiruike Technology Co., Ltd. (Beijing, China); mPEG-SH (Methoxypoly (ethylene glycol)-thiol, MW = 2000) purchased from Beijing Jiankai Technology Co., Ltd. (Beijing, China); and Sodium chloride (NaCl) purchased from Sinopharm Group Chemical Reagent Co., Ltd. (Shanghai, China).

### 2.2. NIR-II Extinction Spectroscopy Measurement System

**Figure 1:**
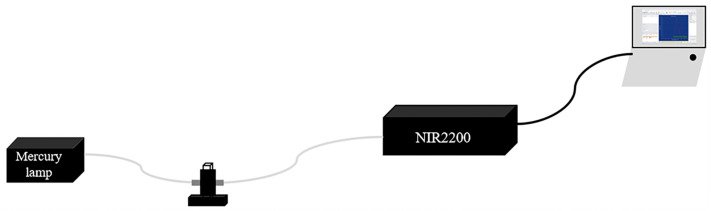
Extinction spectrum test system. A mercury lamp was used as the light source; PG2000 and NIR2200 spectrometers were employed to measure the extinction spectra.

As shown in Figure 1, a mercury lamp was used as the light source; after fixing the sample in a cuvette, the GNRs’ extinction spectra in the ranges of 400–900 nm and 900–1600 nm were measured separately using the PG2000 (PG2000PRO, Ideaoptics, Shanghai, China) and NIR2200 (NIR22px, Ideaoptics, Shanghai, China) spectrophotometers, respectively. The acquired data were subsequently presented on a computer.

### 2.3. NIR-II Dark-Field Microscopic Imaging System

**Figure 2:**
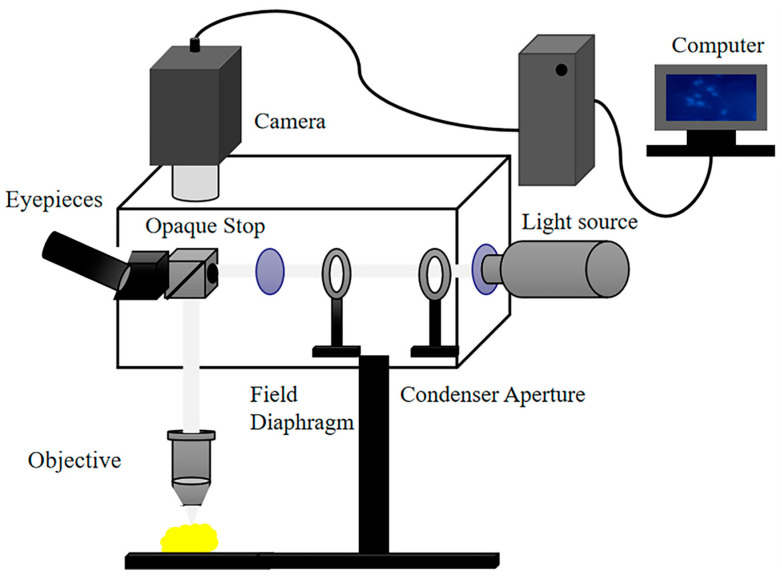
Dark-field microscopy imaging system. The system was sensitive to the 900–1700 nm wavelength range by replacing the commercial visible CCD with an InGaAs camera. In this experiment, different long-pass filters were employed to capture cell images at various wavelengths within this range.

As shown in Figure 2, a dark-field microscope (Eclipse LV150N, Nikon, Tokyo, Japan) was employed in the experiment with a halogen lamp as the light source and an InGaAs camera (SD640, TEKWIN SYSTEM, Shanghai, China) as the photon detector. The light emitted from the lamp passed through an adjustable aperture and a pre-focusing adjustable field stop before achieving Köhler illumination. The imaging system incorporated a dark-field condenser with a dark-field stop, which was a circular shading plate capable of obstructing the central portion of the light beam, allowing only the incident light from the periphery of the circular ring to pass through. After passing through the condenser, the light was inclined from various angles to illuminate the sample surface. A low numerical aperture objective (50×, NA0.8, air) was used in the system, ensuring minimal collection of incident light and primarily collecting the scattered light from the sample. The collected light signal was then used to generate images on a computer.

### 2.4. Calculation of SBR

In order to calculate the intensity of the signal portion in the image, we selected five different regions and obtained the average grayscale values, resulting in IS. The average background intensity IB was similarly obtained using the same method.

The SBR was calculated using the following formula:

$$\mathrm{SBR}=\frac{I_{S}}{I_{B}}$$

### 2.5. One-Pot Synthesis of GNRs

At room temperature, under gentle magnetic stirring, 9.6 mL of deionized water was added to a flask, followed by the addition of HAuCl_4_ solution (0.4 mL, 25 mM). Afterward, a CTAB solution (10 mL, 0.2 mM) was added to the mixture, which turned from pale yellow to orange. After that, AgNO_3_ solution (0.1 mL, 1 M) and HQ solution (1 mL, 0.05 M) were added sequentially. AgNO_3_ facilitated the longitudinal growth of gold nanorods, while HQ reduced Au^3+^ to Au^+^, gradually rendering the solution colorless. When the solution was completely colorless, an ice-cold NaBH_4_ solution (10 µL, 10 mM) was added with vigorous stirring. After one minute, the mixture was placed in a constant temperature drying oven at 30 °C for 24 h to grow the gold nanorods. The solution changed from colorless to dark brown. Excess reactants were removed through centrifugation (9600 rpm, 12 min), yielding GNRs [27].

### 2.6. Polymer Electrolyte Multilayer Coating of GNRs

To remove excess CTAB from the GNRs, the water dispersion of GNRs (10 mL) was centrifuged and washed with water twice. The GNRs were then redispersed in 5 mL of deionized water. PSS solution (30 mM) and NaCl solution (30 mM) were each added (0.5 mL) to the GNR water dispersion. The mixture was vigorously oscillated for 3 min using a vortex oscillator and then stirred for an additional 3 hours. Excess PSS was removed through centrifugation (7200 rpm, 10 min) and washed with water. The resulting product was redispersed in 5 mL of water, yielding GNR-PSS water dispersion. Because the surface of GNRs after PSS characterization is negatively charged and the cell surface is also negatively charged, GNRs need to be modified using PAH. We used a concentration of 10 mM NaCl as a solvent to configure a concentration of 10 mg/mL PAH solution, and we also added 0.5 mL each to the GNR-PSS water dispersion. PAH multilayer modification of GNRs was performed similarly. Finally, the modified GNR-PSS-PAH can be directly incubated with the cells and enter the cells under the action of electrostatic adsorption for subsequent experiments [28].

### 2.7. Preparation of GNR-Based Nuclear Targeting Probes

After centrifugation and washing, m-PEG-SH (400 µL, 10 mg/mL, MW = 2000), membrane localization protein (RGD) (6 μL, 5 mM), and nuclear localization protein (NLS) (25 μL, 5 mM) were added to GNRs (10 mL, 0.1 nM) synthesized using the one-pot method. The mixture was stirred magnetically for 24 h at room temperature. Excess reactants were removed through centrifugation (6000 rpm, 8 min), and the resulting product was redispersed in 1 mL of water.

### 2.8. Characterization of GNRs

The morphologies of GNRs were also studied using a transmission electron microscope (TEM, JEM-1200EX, JEOL, Tokyo, Japan) operating at 80 kV in bright-field mode. The hydrodynamic size distribution was determined through dynamic light scattering (DLS) with a particle size analyzer (Malvern, Zetasizer 3000 HAS, Malvern, UK) at a fixed angle of 90° at room temperature. The zeta potential was determined with the same analyzer (Malvern, Zetasizer 3000 HAS).

### 2.9. Cell Culturing

CT26 cells were used in the experiment and cultured in DMEM supplemented with 10% fetal bovine serum, 1% penicillin–streptomycin, and 1% amphotericin B. Cells were maintained at 37 °C in a humidified atmosphere containing 95% air and 5% carbon dioxide. CT26 cells were seeded in a 24-well plate and incubated in a cell culture incubator for 24 h before sample addition. We used samples of GNR-PSS-PAH and GNR-RGD-NLS with a concentration of 1 nM and added 40 µL to two petri dishes, respectively, and then cultured them with cells for 24 h. We also set up a blank control group.

## 3. Results

### 3.1. Dark-Field Scattering Imaging of Colon Cancer Cells

**Figure 3:**
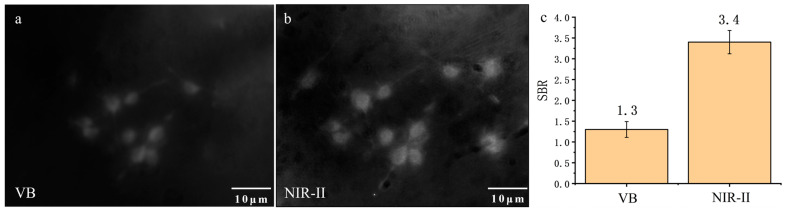
Comparison of imaging effect between visible band and NIR-II band: (**a**) visible band; (**b**) NIR-II window (900–1700 nm); (**c**) shows SBR of the two images.

In our experiments, CT26 cells, a type of colon cancer cell line, were subjected to dark-field scattering imaging in both the visible light spectrum and the NIR-II spectrum (900–1700 nm). As shown in Figure 3a,b, compared to imaging results in the visible spectrum, the imaging quality in the NIR-II spectrum was superior, allowing for clearer visualization of the cell morphology. Upon calculating the SBR for both spectra (Figure 3c), the SBR for the visible spectrum image was 1.3, whereas the SBR for the NIR-II image was 3.4, indicating a higher SBR for the NIR-II image. This confirmed that dark-field imaging of cells in the NIR-II spectrum was more effective. We hypothesized that the improved performance in the NIR-II spectrum was due to the superior anti-scattering ability of longer wavelengths, resulting in fewer signal photons being scattered in the background. Additionally, the visible spectrum is more susceptible to environmental light noise, resulting in a more pronounced background. Additionally, the visible spectrum is more prone to environmental light noise, making background signals more prominent.

### 3.2. Dark-Field Scattering Imaging of Colon Cancer Cells in the NIR-II Window

**Figure 4:**
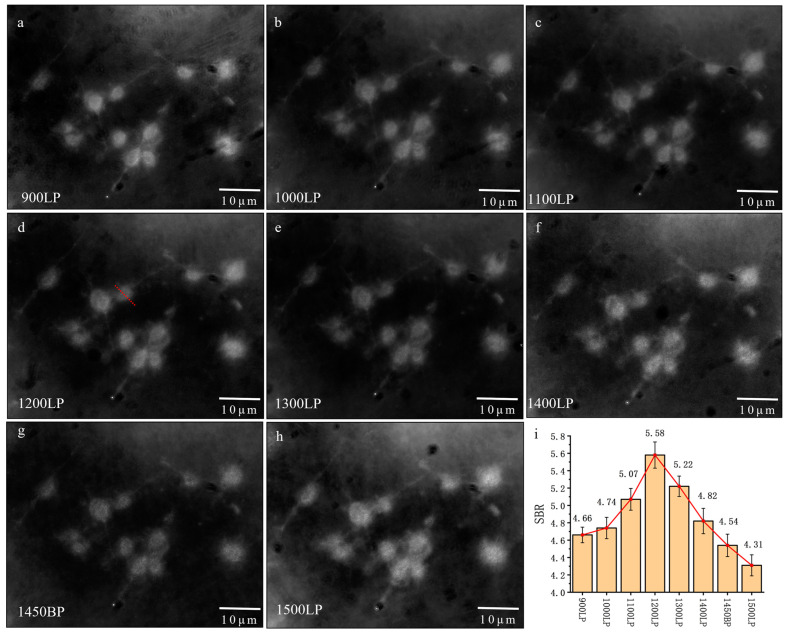
Dark-field imaging of cells in NIR-II window. We added (**a**) 900 LP, (**b**) 1000 LP, (**c**) 1100 LP, (**d**) 1200 LP, (**e**) 1300 LP, (**f**) 1400 LP, (**g**) 1450 BP, and (**h**) 1500 LP to the system; (**i**) shows the comparison of SBR of the images above.

To further investigate the impact of wavelength on imaging, we introduced long-pass filters (LP) ranging from 900 to 1500 nm and a band-pass filter (BP) at 1450 nm into the system, thereby capturing cell images in the corresponding wavelength bands (Figure 4a–h). SBR analysis of the images was performed (Figure 4i). As the imaging wavelength shifted towards longer wavelengths, the SBR gradually increased for bands from 900 to 1700 nm, 1000 to 1700 nm, 1100 to 1700 nm, and 1200 to 1700 nm. However, for bands from 1300 to 1700 nm to 1500 to 1700 nm, SBR exhibited a decreasing trend with the wavelength shift. The cross-sectional signal along the red dashed line of the cell in Figure 4d is presented in Figure S1, indicating a relatively uniform intensity of the cytoplasm and nucleus.

From these results, we observed that as the imaging wavelength shifted towards longer wavelengths, the overall imaging quality improved. However, after the 1200–1700 nm range, the imaging quality began to decline. The reason was speculated to be that as the wavelength increased, the cell’s scattering decreased due to the limited cell size. There is a competition between signal photons and background photons. The decreased scattering resulted in weaker background photons, which improved the imaging quality. Meanwhile, decreased signal photons were harmful to the imaging quality. To leverage the advantages of imaging in the NIR-II window more effectively, we introduced gold nanorods as contrast agents to enhance cell scattering of longer-wavelength light.

### 3.3. NIR-II Dark-Field Scattering Imaging of Cells Assisted by GNRs

**Figure 5:**
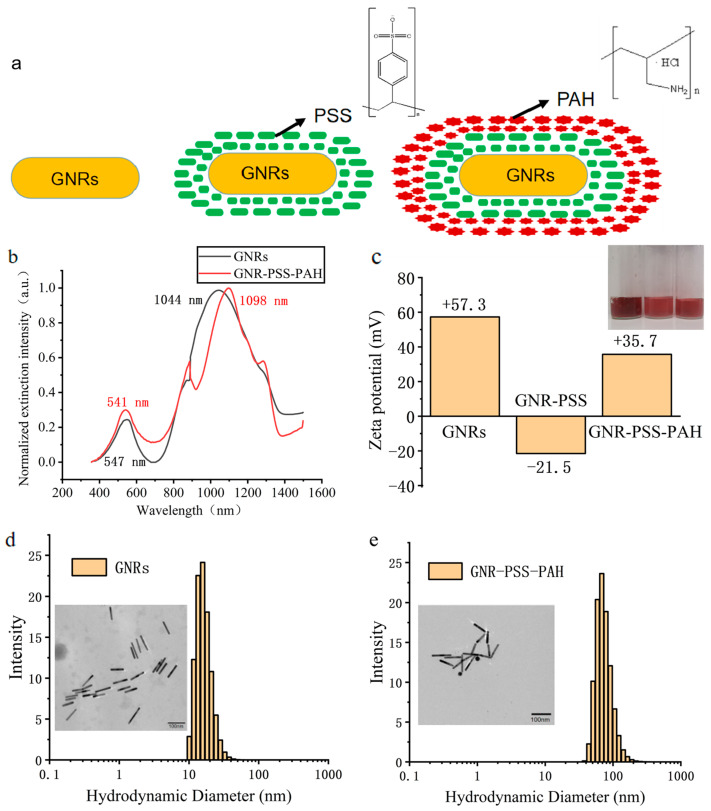
(**a**) Multilayer modification of gold nanorods with polymer electrolyte PSS and PAH. (**b**) Extinction spectra of GNRs and GNR-PSS-PAH. (**c**) Zeta potential of GNRs, GNR-PAA, and GNR-PSS-PAH. (**d**) DLS distribution and transmission electron microscope image (scale 100 nm) of GNRs. (**e**) DLS distribution and transmission electron microscope image (scale 100 nm) of GNR-PSS-PAH.

We synthesized gold nanorods through a one-pot method and performed multilayer modification of the nanorods using polymer electrolytes to enhance their biocompatibility (Figure 5a) [29]. The GNRs synthesized had a longitudinal length of 92.2 ± 8.1 nm and an axial length of 10.2 ± 0.8 nm according to the TEM image, as shown in Figure 5d, and the aspect ratio was about 9.0. The high aspect ratio allowed a redshifted LSPR peak at 1044 nm, which would provide a strong scattering in the NIR-II region. After synthesizing the gold nanorods, we successfully confirmed that they did not aggregate after multiple rounds of centrifugation and washing, indicating a successful coating process. The size of the gold nanorods changed after coating, affecting their localized surface plasmon resonance (LSPR) and resulting in a redshift in the LSPR peak (Figure 5b). Specifically, after multiple layers of coating, the LSPR peak shifted from 1044 nm to 1098 nm, indicating successful PSS and PAH coatings. It can be noticed that two peaks at about 900 nm and 300 nm were not supposed to appear, and they were actually due to the experimental setup and data analysis. Additionally, the zeta potential of GNRs in water dispersion was measured to be +57.3 mV, which changed to −21.5 mV after PSS coating and further increased to +35.7 mV after PAH coating (Figure 5c). These data collectively confirm the successful polymer electrolyte coating on the GNRs’ surfaces. The average hydrodynamic diameter of the GNRs was 16.6 ± 4.6 nm, and that of GNR-PSS-PAH was 75.2 ± 22.3 nm, as shown in Figure 5d,e, respectively. The large difference was because the long chains of polymers would stretch out in water. Considering the measurement model of hydrodynamic diameters was spherical, when gold nanorods with a high aspect ratio were measured, the diameter of equivalent spheres would be much smaller than the real longitudinal length in TEM images. The polymer dispersity index (PDI) of the GNRs and GNR-PSS-PAH was 0.08 and 0.09, which indicated a relatively uniform molecular weight distribution. The TEM size of GNR-PSS-PAH was much smaller than the hydrodynamic diameter because of the drying process for TEM measurement and the low contrast of PSS-PAH in TEM images. All of these results further proved the successful coating of PSS and PAH.

**Figure 6:**
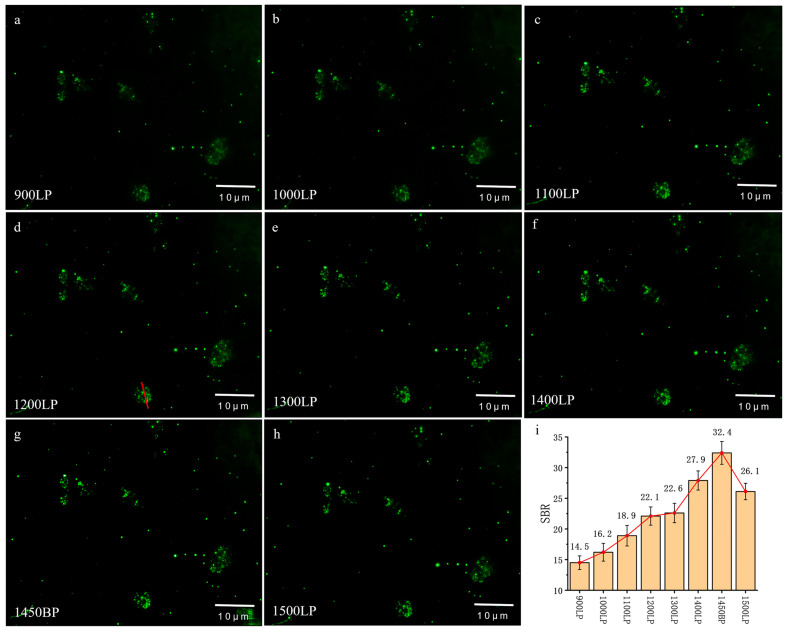
Dark-field imaging of cells in NIR-II window after incubation with gold nanorods. We added (**a**) 900LP, (**b**) 1000LP, (**c**) 1100LP, (**d**) 1200LP, (**e**) 1300LP, (**f**) 1400LP, (**g**) 1450BP, and (**h**) 1500LP to the system; (**i**) shows the comparison of SBR of the image above.

After incubating cells with GNR-PSS-PAH for 24 h, we acquired cell images at different wavelengths, as shown in Figure 6a–h. These images revealed that cell images obtained with GNR labeling were clearer. SBR analysis of the images (Figure 6i) indicated that the cell image SBR gradually increased with a redshift in the imaging wavelength for bands from 900 to 1700 nm, 1000 to 1700 nm, 1100 to 1700 nm, 1200 to 1700 nm, 1300 to 1700 nm, and 1400 to 1700 nm, with the highest SBR in the 1450 nm BP (1425–1475 nm) band, which is 32.4. The cross-sectional signal along the red dashed line of the cell in Figure 6d is presented in Figure S2, indicating a bright cytoplasm and relatively dark nucleus. Images from other fields are shown in Figure S3.

From the above results, it can be found that there was a four to six times improvement in SBR when using GNRs as contrast agents. The best images were observed in the 1450 nm BP (1425–1475 nm) band. This result is attributed to the reduced background noise due to water absorption near 1450 nm.

Moreover, dark regions were observed in the middles of the cells, indicating weaker scattering signals in the central region. This was because GNR-PSS-PAH could only enter the cell cytoplasm via endocytosis but not the cell nucleus. In the following experiments, we modified the gold nanorods with a nuclear localization sequence (NLS) to target the cell nucleus.

### 3.4. NIR-II Dark-Field Scattering Imaging of Nucleus-Specific GNRs

**Figure 7:**
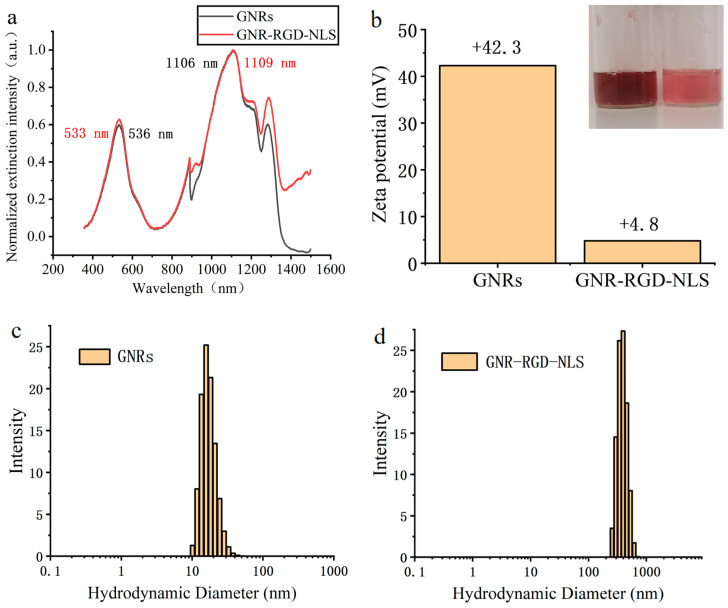
(**a**) Extinction spectra of GNRs and GNR-RGD-NLS. (**b**) Zeta potential of GNRs and GNR-RGD-NLS. (**c**) DLS distribution of GNRs. (**d**) DLS distribution of GNR-RGD-NLS.

We used a nuclear localization sequence (NLS)-specific modification of gold nanorods to target the cell nucleus [30]. Figure 5a,b show the extinction spectra and zeta potential of GNRs and GNR-RGD-NLS, respectively, confirming the successful modification of RGD and NLS on the GNRs’ surface. The LSPR extinction peak of GNRs shifted from 1106 nm to 1109 nm after modification (Figure 7a), with no significant change in the extinction spectrum shape, indicating successful RGD and NLS modifications. It can also be noticed that two peaks at about 900 nm and 300 nm were not supposed to appear, and they were actually due to the experimental setup and data analysis. The zeta potential measurements (Figure 7b) in water dispersion showed that GNRs had a zeta potential of +42.3 mV, which decreased to +4.8 mV after RGD and NLS attachment. The average hydrodynamic diameter of the GNRs was 17.4 ± 4.5 nm, and that of the GNR-RGD-NLS was 388.8 ± 77.3 nm, as shown in Figure 7c,d, respectively. The large difference was because the long chains of polymers would stretch out in water. The polymer dispersity index (PDI) of the GNRs and GNR-RGD-NLS was 0.07 and 0.04, which indicated a relatively uniform molecular weight distribution. These results further confirmed successful surface modifications.

**Figure 8:**
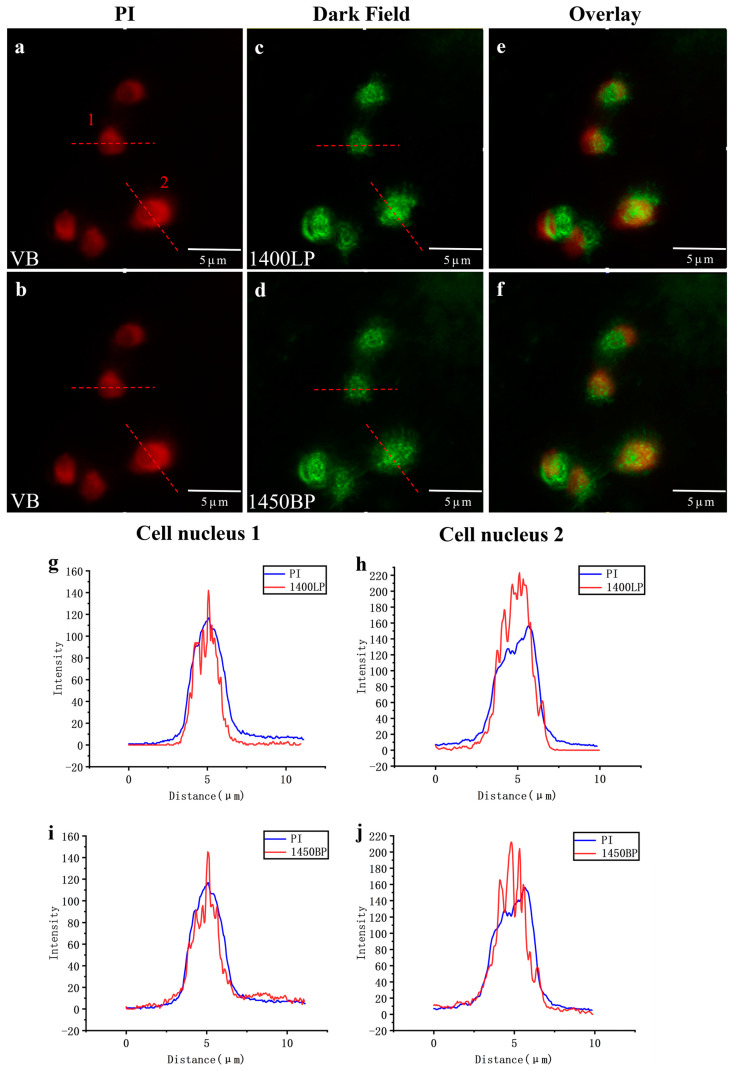
(**a**,**b**) The fluorescent image of cells stained with PI. (**c**) The dark-field image in the band of 1400–1700 nm of cells labeled with GNR-RGD-NLS. (**d**) The dark-field image in the band of 1425–1475 nm of cells labeled with GNR-RGD-NLS. (**e**) The overlapped image of (**a**,**c**). (**f**) The overlapped image of (**b**,**d**). The false color red was for PI and green was for GNRs. (**g**) The signal intensity distribution along the red dashed line of cell 1 in (**a**,**c**). (**h**) The signal intensity distribution along the red dashed line of cell 2 in (**a**,**c**). (**i**) The signal intensity distribution along the red dashed line of cell 1 in (**b**,**d**). (**j**) The signal intensity distribution along the red dashed line of cell 2 in (**b**,**d**).

Subsequently, dark-field microscopy enabled clear visualization of the cell nuclei labeled with GNR-RGD-NLS, exhibiting strong scattering signals in the bands of 1400–1700 nm (Figure 8c) and 1425–1475 nm (Figure 8d). The calculated image SBR values were 35.9 and 26.9, respectively. In addition, we stained the cell nuclei with propidium iodide (PI) to obtain fluorescence images (Figure 8a,b). The overlapped images of dark-field and fluorescence microscopy are shown in Figure 8e,f, with a false color of green for GNRs and red for PI. The overlap image of dark-field and fluorescence images indicated a high colocalization coincidence and the effective and specific labeling of the cell nuclei with GNR-RGD-NLS. Contrary to cells labeled with GNR-PSS-PAH in Figure S2, a bright nucleus was observed for cells labeled with GNR-RGD-NLS, and the cytoplasm was too dark to be seen, as shown in Figure 8g–j. This was because GNR-RGD-NLS could enter the cell nucleus due to the existence of the membrane-penetrating and nuclear localization proteins.

Furthermore, we plotted the signal intensity distribution along the red dashed line of cell 1 in Figure 8a,c, as shown in Figure 8g. The same plotting method was conducted for cell 2, and the result is shown in Figure 8h. It was evident that the dark-field image of cells labeled with GNR-RGD-NLS exhibited stronger signals than that of PI-stained cells. The same plotting method was conducted for cells 1 and 2 in Figure 8b,d, as shown in Figure 8i,j. The result indicated that we also obtained high SBR cell images in the 1425–1475 nm wavelength range (1450 nm BP), commonly referred to as the NIR-IIx window [17]. Considering the significant water absorption and notable photothermal effects of this range, it is rarely used in biological fluorescence imaging. However, from another perspective, thanks to the significant absorption of water, the background noise from scattered photons was further suppressed. In our NIR-II dark-field Microscopy, there was no need to worry about the photothermal effect in this band due to the low photon density of the illuminating source, ensuring the safety of live cells.

## 4. Discussion

Researchers have defined the 900–1880 nm wavelength range as the NIR-II window. Imaging in this range has garnered significant interest in recent years due to its high image contrast and deep imaging capabilities. Dark-field microscopy, known for its affordability, non-destructive sample imaging, and minimal photobleaching, has found wide applications in cellular imaging in the visible band. This paper leverages the advantages of dark-field microscopy in conjunction with NIR-II imaging.

In this article, we primarily introduced a novel technical concept that extended the dark-field scattering microscopy into the near-infrared II region (900–1880 nm). Compared to the traditional visible dark-field scattering microscopy, the advantages of NIR-II dark-field scattering microscopy lie in suppressing the image background and enhancing the signal-to-background ratio, thereby obtaining clearer cell images. These advantages are effective for all types of cells, including but not limited to cancer cells. In this study, we confirmed this with a colon cancer cell line. Despite these advantages, there are still some disadvantages of NIR-II dark-field microscopy. One could be that it is not possible to obtain the bright field image of the same area at the same time as the contrast. In addition, the signal intensity of the samples decreases as the background intensity is suppressed. Therefore, we proposed the strategy of introducing gold nanorods as the scattering agents to enhance the signal intensity in the regions of interest. Compared to the traditional dark-field scattering imaging, our approach has a higher signal-to-background ratio and better specificity. By employing different modifications on the gold nanorods, we could image the cytoplasm and nucleus of cells separately, as we have demonstrated in this article. In future work, we can explore more modification methods for gold nanorods to obtain targeted images of specific organelles, thereby broadening the application fields of NIR-II dark-field microscopy.

## 5. Conclusions

We optimized a traditional dark-field imaging system to perform dark-field imaging of cells in the NIR-II range. Compared to images in the visible band, images in the NIR-II window exhibited reduced background noise, clearer cell contours, and increased SBR as the wavelength increased. To further increase the contrast of the cells, GNRs with an LSPR extinction peak in the NIR-II band were synthesized and modified with PSS and PAH. Dark-field images of GNR-PSS-PAH-labeled cells were most favorable in the 1400–1700 nm wavelength range with an SBR of 32.4, which was approximately six times higher than images without GNRs. In those images, the cytoplasm was bright while the nucleus was rather dark. Then, we modified GNRs with nuclear targeting proteins and obtained the nuclear-specific probe GNR-RGD-NLS. Images of GNR-RGD-NLS-labeled cells were obtained in both 1400–1700 nm and 1425–1475 nm bands, displaying well-defined nuclear boundaries. The colocalization coincidence with nuclear dye PI was very high, and the image in 1400–1700 nm had a high SBR of 35.9. These results underscored positive implications of our NIR-II dark-field microscopy in suppressing the image background and enhancing the signal-to-background ratio, thereby obtaining clearer cell images.

## Data Availability

The data that support the findings of this study are available from the corresponding author (Wen Liu) upon reasonable request.

## Supplementary Materials

The following supporting information can be downloaded at: https://www.mdpi.com/article/10.3390/biology12111391/s1, Figure S1: The cross-sectional signal along the red dashed line of the cell in Figure 4d, indicating a relatively uniform intensity of the cytoplasm and nucleus; Figure S2: The cross-sectional signal along the red dashed line of the cell in Figure 6d, indicating a bright cytoplasm and rather dark nucleus; Figure S3: Images from other fields. (a–c): Images of cells incubated with GNR-RGD-NLS.
