# Supplementary material for: Gold-Nanorod-Assisted Live Cell Nuclear Imaging Based on Near-Infrared II Dark-Field Microscopy
Source: Biology (Basel). 2023 Oct 31;12(11):1391. doi: 10.3390/biology12111391 (PMC10669354; doi:10.3390/biology12111391)
Supplement: Supplementary file 1 [file biology-12-01391-s001.zip › biology-2658579-supplementary.pdf]

Supplementary Materials:

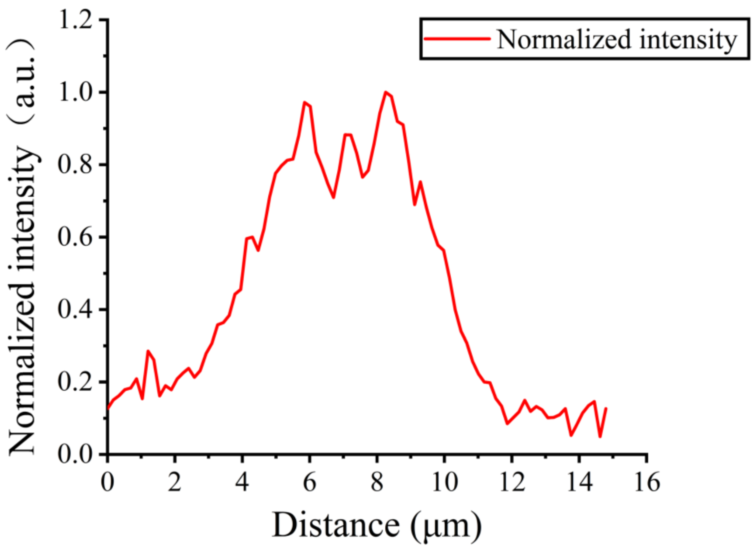

Figure S1: The cross-sectional signal along the red dashed line of the cell in Figure 4d, indicating a relatively uniform intensity of the cytoplasm and nucleus.

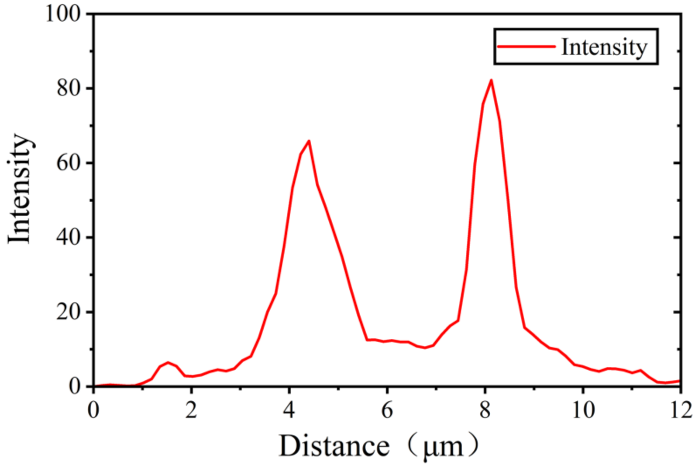

Figure S2: The cross-sectional signal along the red dashed line of the cell in Figure 6d, indicating a bright cytoplasm and rather dark nucleus.

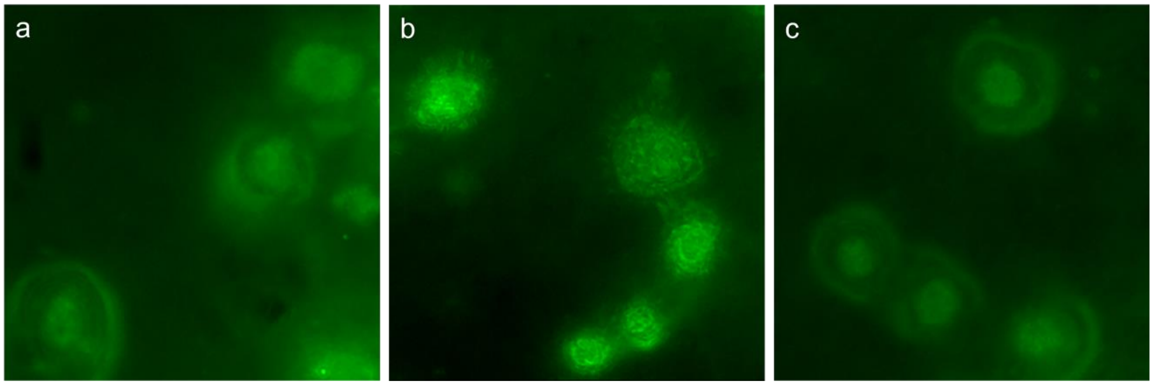

Figure S3: Images from other fields. (a),(b),(c): Images of cells incubated with GNR-RGD-NLS.
